# Local Emergence of a del HV69-70 SARS-CoV-2 Variant in Burgundy, France

**DOI:** 10.3390/pathogens11020124

**Published:** 2022-01-20

**Authors:** Hélène Giraudon, Mohand Djemai, Christelle Auvray, Alexis de Rougemont, Gaël Belliot, Jean-Baptiste Bour, Catherine Manoha

**Affiliations:** 1 Department of Microbiology Virology Laboratory, Dijon Bourgogne University Hospital, 21070 Dijon, France; helene.giraudon@chu-dijon.fr (H.G.); christelle.auvray@chu-dijon.fr (C.A.); alexis.de-rougemont@u-bourgogne.fr (A.d.R.); gael.belliot@chu-dijon.fr (G.B.); Jean-Baptiste.Bour@u-bourgogne.fr (J.-B.B.); 2 Department of Biology, Montceau-les-Mines Hospital, 71300 Montceau-les-Mines, France; MoDJEMAI@ch-montceau71.fr

**Keywords:** SARS-CoV-2, outbreak, deletion, elderly, nursing home

## Abstract

In the autumn of 2020, a short-lived epidemic of a spike del69-70 deletion variant of SARS-CoV-2 was identified, with most cases (*n* = 95) found in Montceau-les-Mines, France. This spike gene target failure (SGTF) variant spread quickly in nursing homes. The Alpha variant, which also harbors this deletion, appeared in Burgundy in January 2021 after the disappearance of the Montceau-les-Mines del69-70 variant. Our findings illustrate the risk of the fast spread of geographically isolated variants and reinforce the need for the continuous tracking of outbreaks. In some cases, these studies may reveal emerging variants that affect public health or vaccine development.

## 1. SARS-CoV-2 with a 69–70 Spike Deletion 

Some SARS-CoV-2 variants are of particular concern because they have a clear impact on public health due to their increased transmissibility, increased infection severity or immune escape; these are referred to as “variants of concern” (VOC). Other variants are classified as variants of interest (VOI) or variants under monitoring (VUM) for those detected as signals to follow. The misamplification of the S gene using Applied Biosystems™ TaqPath™ COVID-19 RT-PCR Kit from ThermoFisher Scientific was shown to be fully concordant with the deletion of H69/V70 (del HV69-70) in a study in the area of Lyon, France [1]. We report here on a del HV69-70 strain that emerged in Burgundy in autumn, 2020, before vaccination availability, and spread among nursing homes. This variant was present in as many as 21.1% of SARS-CoV-2-positive samples in the city of Montceau-les-Mines, Burgundy, France. Although the impact of del HV69-70 on public health has not been formally demonstrated, its virological, clinical and epidemiological characteristics deserve attention.

## 2. Continued Increase in the Number of SARS-CoV-2 Cases until the Lockdown Was Implemented

Routine SARS-CoV-2 surveillance was performed at a community-based testing platform in the virology laboratory of Dijon University Hospital using the Applied Biosystems™ TaqPath™ COVID-19 RT-PCR Kit (ThermoFisher Scientific, Waltham, MA, USA) that includes the open reading frame (ORF) 1ab, the spike (S) and nucleocapsid (N) gene targets. A sample was considered positive when amplification was detected from at least two targets with a Ct value < 37. In total, 14519 samples were screened for SARS-CoV-2 over the 10-week study period (weeks 38 to 47), of which 2022 (13.9%) were positive. Most samples (97.7%) came from the hospital in Dijon and four public hospitals in the same department (Auxerre, Beaune, Langres and Montceau-les-Mines); 98.2% of positive samples came from these five hospitals, which are all located in the FRC1 statistical region according to the European Nomenclature of Territorial Units for Statistics (NUTS) level 2. Patients positive for SARS-CoV-2 were a median age of 48 years.

**Figure 1:**
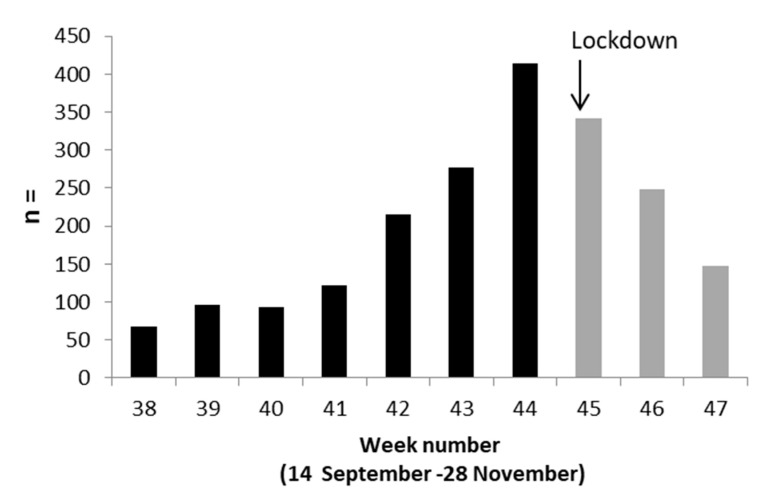
SARS-CoV-2 positives samples per week in 2020.

France went into its first lockdown on 17 March until 10 May, 2020. Afterwards, there was a significant increase in the number of positive SARS-CoV-2 tests over time until a second lockdown was implemented from 30 October to 15 December, 2020 (weeks 45 to 50) (Figure 1). The first lockdown was shown to reduce the reproduction number from 2.90 to 0.67 (77% reduction) [2]. Our results show that the second French lockdown was also immediately effective seeing as the number of positive samples decreased continuously from week 45 to the end of the study.

## 3. S-Gene Target Failure RT-PCR Assay Is Associated with del HV69-70

The analysis of the RT-qPCR results showed that 108 out of 2022 (5.3%) positive tests from 30 October until 15 December 2020 had no or low amplification of the S gene, while ORF1ab and N targets were correctly amplified (SGTF samples) (Figure S1). SGTF samples reveal a HV69-70 deletion in the spike protein as shown by Bal et al. [1]. We explored the misamplification using whole genome sequencing in 13 samples collected throughout the study period. The CleanPlex^®^ SARS-CoV-2 Flex panel from Paragon Genomics, Hayward, CA, USA, with dual indexing was used for library preparation. Whole genome sequencing was performed on an Illumina MiSeq (Illumina, San Diego, CA, USA) platform (300 cycles, paired-end reads). Data analysis was performed by Exatype (Hyrax Biosciences, Cape Town, South Africa). All sequencing reads were mapped to the SARS-CoV-2 genome (reference sequence (Wuhan-Hu-1, NC_045512.2) and the sequences were submitted to GISAID (https://www.gisaid.org/, accessed on 20 December 2021; accession numbers). All of the sequences included a 6-nucleotide deletion (nt 21765–21770) within the spike protein in 100% of the reads, which confirmed the deletion of a histidine and a valine at the amino acid positions 69 and 70 in the S1 subunit (Figure S1c). In addition to the del HV69-70, two substitutions, N439K and D614G, were found within the spike protein, as well as two amino acid substitutions in ORF1a and five amino acid substitutions in ORF1b. In two of them, an additional spike mutation A688V was detected, a substitution located three amino acids downstream from the furin cleavage site.

Our variant is characterized by the deletion of HV69-70 and the substitutions of both N439K and D614G within the spike protein. All 13 sequenced samples had the N439K substitutions, which means that a large majority of samples had the N439K (100% (75–100%)). The Nextstrain Clade 19B emerged in late 2019, but early in the pandemic, it was overtaken by clades 20A, 20B and 20C [3,4], which harbor the D614G substitution believed to increase viral transmission, but not pathogenicity [5,6], a conclusion that is supported by experimental evidence [7].

From June to November, 2020, in Denmark, a large number of minks became infected with a SARS-CoV-2 variant harboring the deletion H69 and V70 [8]. The del69-70 of our variant is the only common point with the mink cluster-5 variant, suggesting independent occurrences. Del HV69-70 was shown to increase spike cleavage and compensate for RBD mutations [9]. A recent analysis highlighted the potential for enhanced transmissibility of viruses with deletions in the N-terminal domain, including del HV69-70 [10]. Moreover, the N439K mutation, which is located in the RBD, may lead to a similar clinical spectrum but affected fitness, as shown in antibody evasion assays [11]. This mutation, which was first sampled in Scotland, was later found in Romania and Norway, forming a second lineage [11,12]. The N439K mutation then frequently arose independently, spreading in Europe and beyond from August, 2020, onwards.

## 4. Localized High Proportion of del HV69-70 Detected in RT-PCR Data

**Figure 2:**
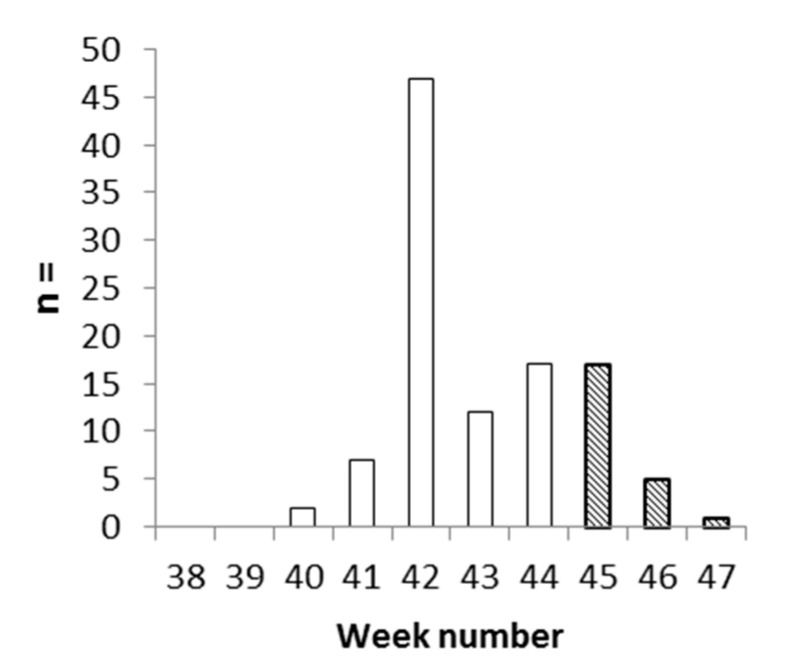
SARS-CoV-2 SGTF positive samples per week. Hatched bars: lockdown period.

**Figure 3:**
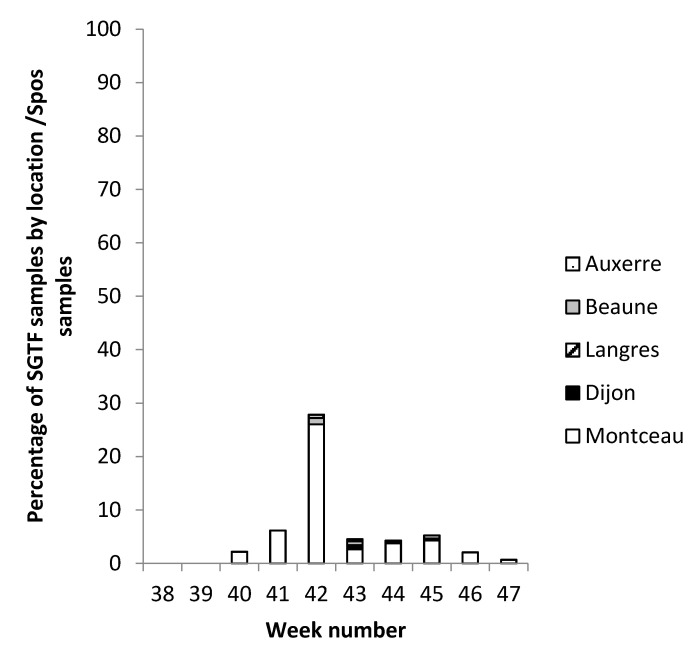
Geographical location of SGTF samples/week. The geographical location is expressed as a percentage of SGTF samples by location/Spos samples per week.

There was an increase in SGTF cases over time from weeks 40 to 45, with a peak during week 42 (Figure 2). The proportion SGTF/positive for the S gene (Spos) ranged from 0% (weeks 38–39) to a prevalence of around 6% from weeks 41 to 45 and peaking at 27.2% during week 42. Most of the SGTF samples originated from Montceau-les-Mines (88%) and only sporadic cases were seen in the four other sites (Figure 3). This was not due to a bias in the recruitment between the different hospitals, but results of a real difference in the emergence of the variant. The local proportion (mean of 21.1% over the study period) increased to 66.7% in week 42, despite the good observance of preventive measures, suggesting that the variant was highly transmissible. SGTF cases were mainly found among patients from nursing homes. In this elderly population, this variant was associated with a possible greater transmissibility compared to the Spos virus. Vigilance may thus be required for the surveillance of variants including del HV69-70 and/or N439K. In the city of Lyon, SGTF was detected in 0.6% of positive samples, but there was no significant increase over time; the monthly proportion fluctuated from 0% to 2.8% from weeks 32 to 51 in 2020 [1], which does not support the elevated transmissibility of SGTF strains. However, three quarters of the documented SGTF cases in Lyon had a HV69-70 deletion, but did not include the N439K substitution. The association of del HV69-70 and N439K might be associated with increased transmissibility.

We focused on the epidemic in Montceau-les-Mines. The del HV69-70 variant was first found in three health care workers at the end of September, 2020 (week 38–39). Two of the three health care workers were from the same family, and they were also related to two other individuals, one other health care worker and one person living in a nursing home. Both become infected with the SGTF variant in mid-October.

**Figure 4:**
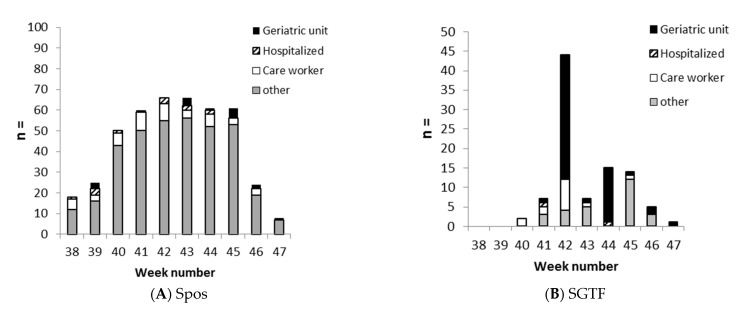
Groups of patients from Montceau-les-Mines.

The SGTF variant was almost exclusively and notably found in nursing homes even though it was the second wave of SARS-CoV-2 epidemic and stringent hygiene policies had been in place for a long time in order to limit the risk of contamination, in particular during personal interaction with local staff. Two nursing homes located 5 km (3 miles) from Montceau-les-Mines were impacted by the variant. Out of the 95 cases of SGTF, 50 cases were from nursing homes; out of the 444 cases of Spos, only 17 were from nursing homes (3.8%). This may suggest that this particular variant is more transmissible in the elderly (Figure 4).

Patients infected with Spos SARS-CoV-2 were younger than patients infected with SGTF SARS-CoV-2 (mean age: Spos group 48.7 ± 2.6% (43.5–54%); SGTF group 66.4 ± 2.75% (60.9–71.8%); Kruskal–Wallis (KW) test: *p* < 0.0001). The sex ratio (SR) of 0.38 for SGTF group was significantly different from that of the Spos group (SR = 0.95); females in the SGTF group: *n*/*n* total= 69/95; 72.6% (62.5–81.3%); females in the Spos group: *n*/*n* total = 228/444; 51.3% (46.6–56.1%); χ^2^ test: *p* < 0.001. Female sex was more frequently associated with SGTF infection than with Spos infection.

The distribution of asymptomatic infectious episodes by age (0–60) was similar in patients for both S groups. However, among patients older than 60 years, there were significantly more asymptomatic patients in the SGTF group than in the Spos group (SGTF *n* = 42/59, 71.1% (57.9–82.2%); Spos *n* = 60/138, 43.5% (35.1–52.2%); χ^2^ test: *p* < 0.001, suggesting that the SGTF variant could be less severe in older patients (Figure S2). However, as screening is conducted immediately after identification of a first positive case in nursing homes, the sampling approach may explain the large number of asymptomatic patients. The lower severity we observed following the SGTF infection in the 60–80 age group needs to be confirmed.

The cycle threshold (Ct) was used as an inverse proxy for viral load, with lower Ct correlating with higher viral load. The viral load in the SGTF group was found to be higher (median (N) Ct = 16.1) when compared to the Spos group (-median (N) Ct = 23.6) and may be responsible for the transmissibility of the SGTF variant. The Ct values are presented in Table S1.

Considering the rapid spread observed in nursing homes, our report suggests that elderly people are susceptible to infection by SGTF SARS-CoV-2. Higher viral load, but also bias such as gender, age, high level of sampling and health care interactions, may have played a role in the viral spread. When the community incidence of SARS-CoV-2 increases, care home residents are susceptible to outbreaks [13,14]. Recently, a metadata analysis of SARS-CoV-2 genomes was performed with respect to genomic clades and geographic, age and gender distributions [15]. The analysis showed a higher prevalence of severe or deceased cases among the male patients. Furthermore, severe disease or death were more frequent in the elderly than in adults and children [15,16].

The variant from Montceau-les-Mines is derived from clade 20A and defined by the del HV69-70. This characteristic is notably shared with the VOC B.1.1.7 (Alpha variant), which first emerged in the United Kingdom. Seeing that the first detection of the Alpha variant in France was reported on 20 December, 2020, at the virology laboratory of Tours University Hospital (GISAID accession number: EPI_ISL_735391) and was only detected in Burgundy on 14 January, 2021, its circulation is irrelevant for our study period. From mid-January, we observed a dramatic increase in Alpha variant contaminations in our region. The 13 SARS-CoV-2 strains analyzed in this study belong to the clade 20A and the lineage B.1.258. Genomic epidemiology of SARS-CoV-2 in 2020 was estimated by analyzing two Europe-focused subsamplings performed using Nextstrain [17]. From December, 2019, to 13 September, 2020, before the 10-week study period, the 202 sampled sequences of 3868 genomes did not include any variant similar to our variant. The Nextstrain clade 20B included 41% of the strains (92 out of 202 European strains). Strains belonging to clades 20A were detected in 31% (62 out of 202) of the strains and to 19A in 7% (14 out of 2020) (Figure S3). The most prevalent Pango lineages in the analyzed strains were B.1 (28%) and B.1.1 (22%). Among the selected sequences from Nextstrain during the study period, from weeks 38 to 47, 2020 (*n* = 86/3738), nine had the 6-nucleotide HV deletion and most were found in Switzerland and Liechtenstein. The clade 20A took over and the dataset revealed an increase in the variant B.1.258 with a del69-70 to 10.55% (4.9–18.9%). A phylogenetic tree representative of the viral variability circulating at that period in Europe is available as a supplementary document (Figure S4).

Between the waves of SARS-CoV-2 (Alpha to Delta, and then, to Omicron), many variants have risen and fallen [18,19]. The two-amino acid 69–70 deletion has arisen independently several times [9]. Most deletions appear to have arisen and been retained in replication-competent viruses. It should be noted that the Alpha variant requires the deletion of HV69-70 for optimal spike cleavage and infectivity [10]. The Omicron variant is the novel VOC [20]. The lineage BA.1, which is spreading across the world, is characterized by numerous amino acid changes in the spike protein, including the deletion HV69-70 [21]. 

We report the outbreak of a SARS-CoV-2 variant circulating locally in France that totally vanished after the second lockdown. This type of sudden appearance and fast spread of an original variant could potentially be observed anywhere and include strains of worrying virulence or vaccine escape variants. This work emphasizes the need for the molecular surveillance of SARS-CoV-2 in order to track the emergence of new variants with potential epidemiological or pathophysiological consequences. 

## Data Availability

All the data presented in the study are included in the article. Further enquires can be directed to the corresponding author.

## Supplementary Materials

The following supporting information can be downloaded at: https://www.mdpi.com/article/10.3390/pathogens11020124/s1, Figure S1: Amplification curves for the three targets included in Applied Biosystem™ TaqPath™ COVID-19 RT-PCR kit.; Figure S2: Percentage of Symptomatic patients according to age for Spos and SGTF samples from Montceau-les-Mines, Burgundy, France; Figure S3: Viral variability in Europe; Figure S4.: Phylogenetic tree representing the relationship among the 13 SARS-CoV-2 sequences from Burgundy and 86 randomly selected genomes collected during the study period in Europe; Table S1: Viral load.
