# Supplementary material for: Local Emergence of a del HV69-70 SARS-CoV-2 Variant in Burgundy, France
Source: Pathogens. 2022 Jan 20;11(2):124. doi: 10.3390/pathogens11020124 (PMC8875006; doi:10.3390/pathogens11020124)
Supplement: Supplementary file 1 [file pathogens-11-00124-s001.zip › S1 pathogens .pdf]

a)

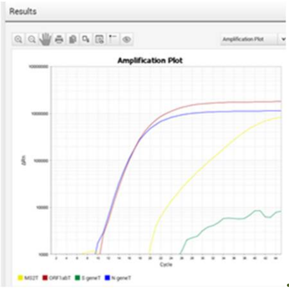

b)

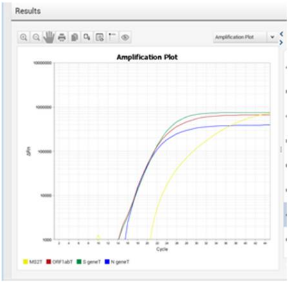

c)

```
 W   F   H   A   I        S   G   T
*************        ***********
tggttccatgcta------tctctgggacc  SGTF del 69-70
tggttccatgctatacatgtctctgggacc  Spos
 W  F   H   A   I  H   V   S   G   T
```

Figure S1 Amplification curves for the three targets included in Applied Biosystem™ TaqPath™ COVID-19 RT-PCR kit.

a) SGTF sample, SARS-CoV-2 with nucleotide deletion 21765–21770 in the spike gene, amplification curves (Ct= 10) obtained for the two targets (ORF1ab, N), MS2 is the internal control (Ct =20)

b) Spos sample, amplification curves (Ct= 14) obtained for the three targets (ORF1ab, N, S)

MS2 is the internal control (Ct= 20)

c) Sequence alignment

The sequence, nt 21748-21780, within the S gene, which lacks 6 nucleotides (i.e. 21765–21770 deletion) is in the upper part of the alignment and the reference sequence (Wuhan-Hu-1) is in the lower part. Amino acids encoded by the nucleotide sequence are shown. The S gene 21765–21770 deletion results in the deletion of amino-acid residues H69 and V70 in the S protein.
