# Supplementary material for: Local Emergence of a del HV69-70 SARS-CoV-2 Variant in Burgundy, France
Source: Pathogens. 2022 Jan 20;11(2):124. doi: 10.3390/pathogens11020124 (PMC8875006; doi:10.3390/pathogens11020124)
Supplement: Supplementary file 1 [file pathogens-11-00124-s001.zip › S2 pathogens .pdf]

A)

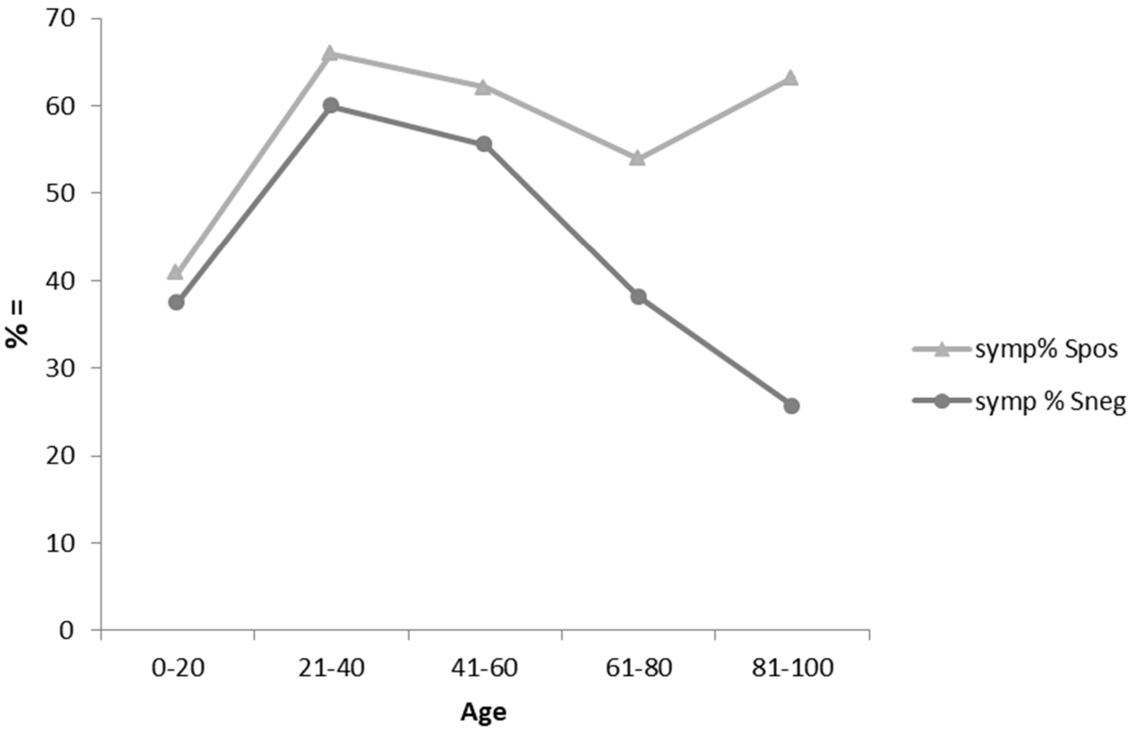

B)

	Spos (n=)		SGTF (n=)	
Age	Symptomatic	Asymptomatic	Symptomatic	Asymptomatic
**0-20**	36	52	3	5
**21-40**	62	32	6	4
**41-60**	72	44	5	5
**61-80**	54	46	8	13
**81-100**	24	17	9	29

Figure S2 Percentage of Symptomatic patients according to age for Spos and SGTF samples from Montceau-les-Mines, Burgundy, France

A) Percentage of symptomatic patients/ total

B) Number of symptomatic and asymptomatic patients and viral load in Spos and SGTF groups
