# Supplementary material for: Local Emergence of a del HV69-70 SARS-CoV-2 Variant in Burgundy, France
Source: Pathogens. 2022 Jan 20;11(2):124. doi: 10.3390/pathogens11020124 (PMC8875006; doi:10.3390/pathogens11020124)
Supplement: Supplementary file 1 [file pathogens-11-00124-s001.zip › S3 pathogens.pdf]

A)

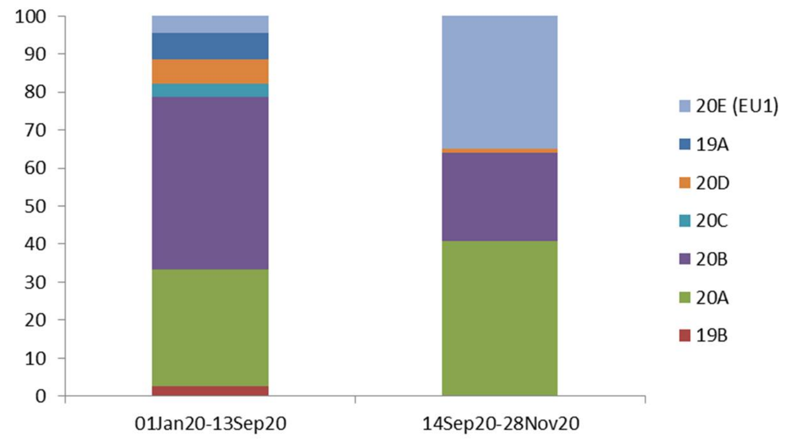

B)

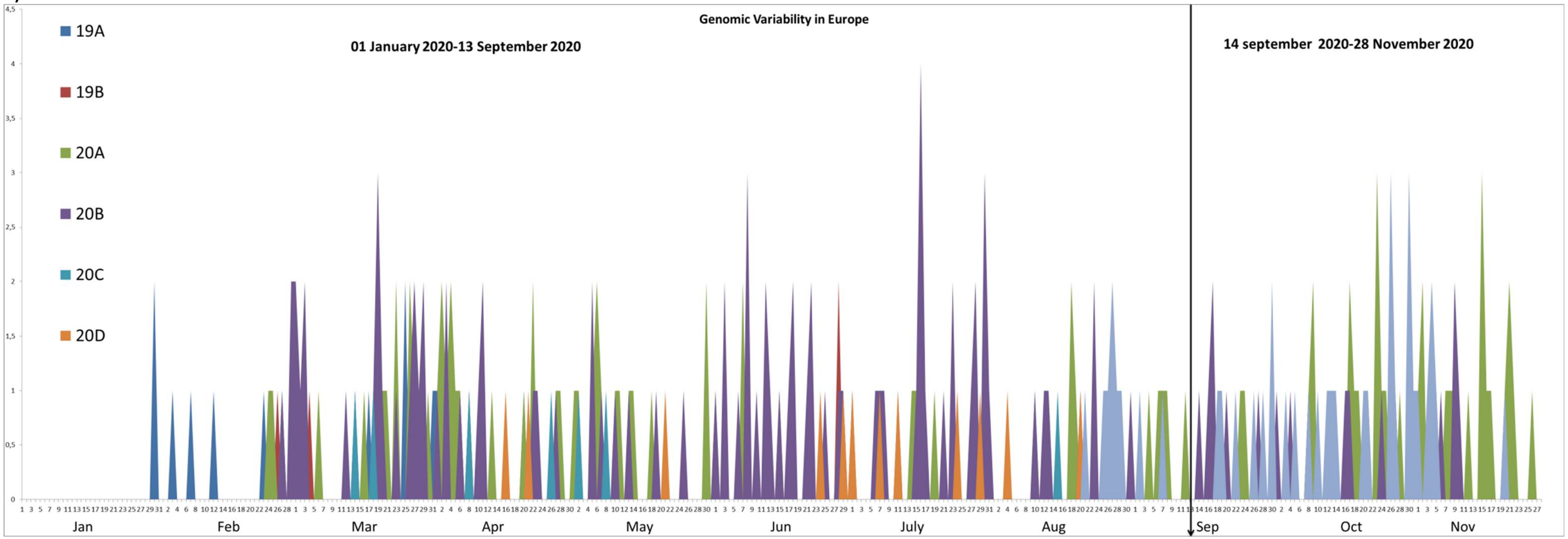

Figure S3 Viral variability in Europe

A. Percentage distribution of clades among the randomly selected European SARS-CoV-2 strains, from December 2019 to mid-September 2020, before the study period and from mid-September to end of November 2020 , during the study period.
B. Decrease in genomic variability over time
