# Supplementary material for: Local Emergence of a del HV69-70 SARS-CoV-2 Variant in Burgundy, France
Source: Pathogens. 2022 Jan 20;11(2):124. doi: 10.3390/pathogens11020124 (PMC8875006; doi:10.3390/pathogens11020124)
Supplement: Supplementary file 1 [file pathogens-11-00124-s001.zip › S4 pathogen tree1401 .pdf]

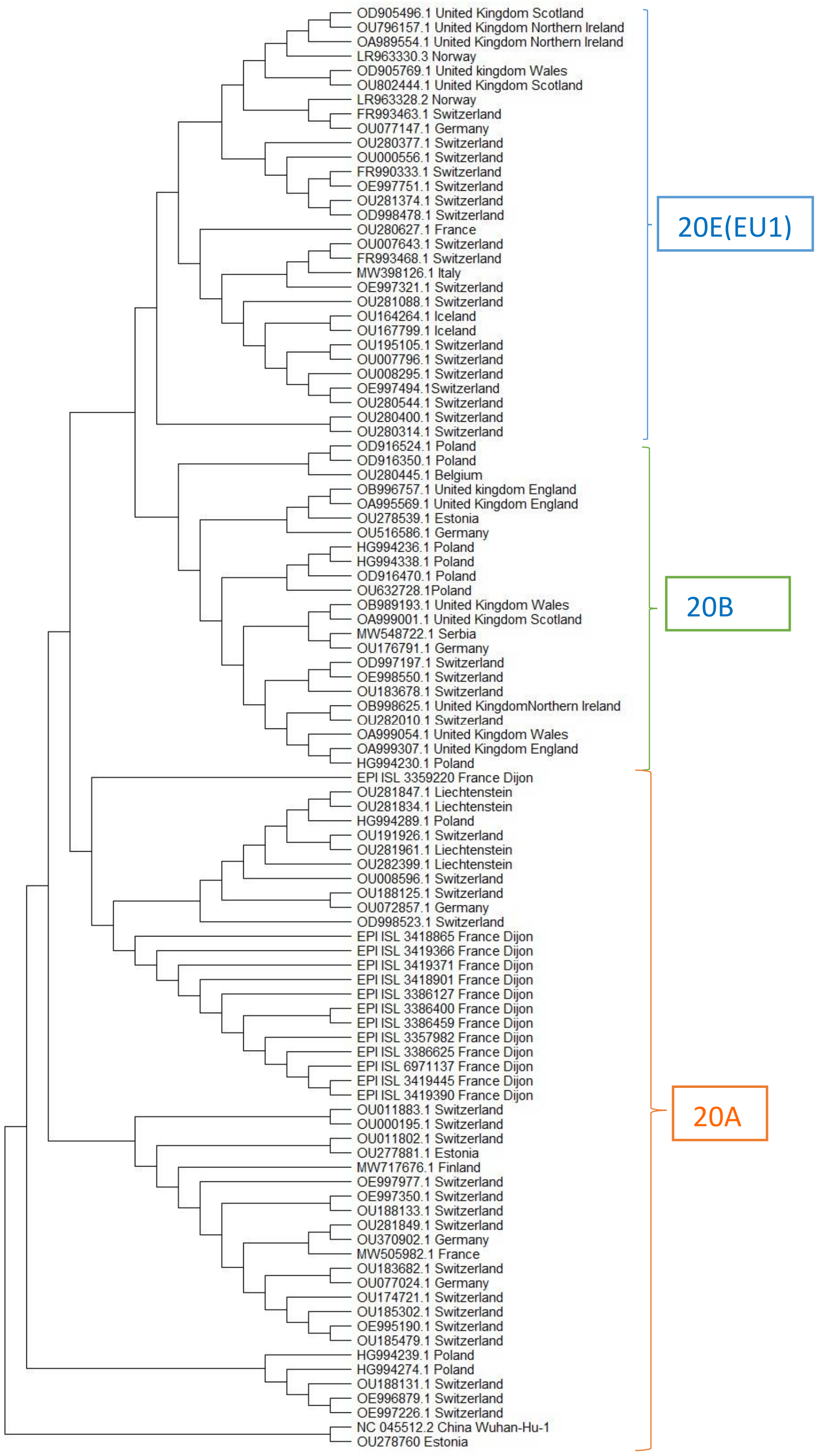

Figure S4. Phylogenetic tree representing the relationship among the 13 SARS-CoV-2 sequences from Burgundy and 86 randomly selected genomes collected during the study period in Europe

Using MegaX, genome sequences were first aligned with CLUSTAL and the relationship tree was established via maximum likelihood, with a bootstrap value of 500, followed by a rooting on Wuhan-Hu-1 sequence. SARS-CoV-2 strains randomly selected by Nextstrain are indicated by genbank accession number and country; SARS-CoV-2 strains presented in this study are indicated by GISAID accession number, Country (France) and town of RT-PCR analysis (Dijon): EPI_ISL_3357982;EPI_ISL_3359220; EPI_ISL_3386459; EPI_ISL_3386625; EPI_ISL_3386127 hCoV-19/France/VIP-1510/2020; EPI_ISL_3386400; EPI_ISL_3419445; EPI_ISL_3419366 EPI_ISL_3419390;EPI_ISL_3419371; EPI_ISL_3418865; EPI_ISL_3418901; EPI_ISL_6971137
