# Supplementary material for: Local Emergence of a del HV69-70 SARS-CoV-2 Variant in Burgundy, France
Source: Pathogens. 2022 Jan 20;11(2):124. doi: 10.3390/pathogens11020124 (PMC8875006; doi:10.3390/pathogens11020124)
Supplement: Supplementary file 1 [file pathogens-11-00124-s001.zip › Table S1.pdf]

	Spos		SGTF		**_p_- value**
	n=	mean Ct	n=	mean Ct	
	444	**23**	95	**18,7**	p<0,000
**Age (years)**					
0-20	89	**25,3**	8	**27,7**	
20-60	213	**23**	26	**18,4**	p<0,01
61-100	142	**21,4**	61	**17,8**	p<0,01
**Symptomatology**					
Symptomatic patients	248	**20,9**	32	**18,6**	
Asymptomatic patients	188	**26**	55	**19**	p<0,000

Table S1. Viral load

The Ct values associated with Spos group and SGTF group were compared using 1-way analysis' test or Kruskal-Wallis' test. The viral load in the SGTF group was higher when compared to the Spos group. Of note, the differences were not statistically significant for 0-20 age group. Viral loads were higher for asymptomatic patients in the SGTF group compared to asymptomatic patients in the Spos group.
